# Political repression motivates anti-government violence

**DOI:** 10.1098/rsos.221227

**Published:** 2023-06-14

**Authors:** Henrikas Bartusevičius, Florian van Leeuwen, Michael Bang Petersen

**Affiliations:** ^1^ Peace Research Institute Oslo, Oslo, Norway; ^2^ Department of Social Psychology, Tilburg University, Tilburg, Netherlands; ^3^ Department of Political Science, Aarhus University, Aarhus, Denmark

**Keywords:** aggression, repression, political violence, collective action, anti-government protest, human rights

## Abstract

We examined whether political repression deters citizens from engaging in anti-government behaviour (its intended goal) or in fact motivates it. Analyses of 101 nationally representative samples from three continents (*N* = 139 266) revealed a positive association between perceived levels of repression and intentions to engage in anti-government violence. Additional analyses of fine-grained data from three countries characterized by widespread repression and anti-government violence (*N* = 2960) identified a positive association between personal experience with repression and intentions to engage in anti-government violence. Randomized experiments revealed that thoughts about repression also motivate participation in anti-government violence. These results suggest that political repression, aside from being normatively abhorrent, motivates anti-repressor violence.

## 1. Introduction

The majority of the world's population faces threats of political repression [1]. Such repression can range from surveillance and harassment of ordinary citizens to torture and ‘disappearances’ of opposition activists. The goal of political repression is to quell the opposition that is contesting power and to prevent citizens from engaging in anti-government activities or having anti-government views [2]^1^. But how do people react to such coercion?

Political repression enforces social hierarchies and group-based discrimination [3], and perceptions of discrimination are associated with lower well-being [4]. Political repression also undermines people's sense of power, and lack of power intensifies negative emotions, weakens positive ones [5–8], strengthens behavioural inhibitions [9] and impedes goal pursuit [10,11]. Political repression—particularly if violent—also generates psychological distress, often leading to depression and/or post-traumatic stress disorder [12,13], which in turn are associated with apathy and inactivity. Hence, research on the psychological consequences of repression, or other variables associated with repression, suggests that political repression potentially deters anti-government behaviour.

However, this conjecture would go against the main proposition of reactance theory: restrictions of freedom of choice often elicit motivations to engage in freedom-restoring behaviours [14]. Furthermore, insurgency researchers have long theorized that violence against civilians incites, rather than suppresses, armed insurgencies or rebellions [15,16]. Some accounts also theorize heterogeneous effects: coercion against civilians likely elicits revenge motivations—but people may or may not act on such motivations depending on the costs of doing so [15,16].

Empirically, only a handful of individual-level studies have directly analysed how repression affects people's motivations to engage in collective actions against the repressors. Some studies have analysed covert or individual behaviours, such as redefining one's identity, using humour, or other forms of psychological resistance to repression [17–19]. Other studies have examined collective actions in repressive contexts, yet this research has focused on the psychological antecedents of participation in collective actions, such as risk perceptions, not on repression as the driving cause [20,21]. The few existing studies that have analysed repression as the driving cause relied on single-country samples and analysed dissent as an aggregate category, without distinguishing between non-violent and violent forms [22,23]. The larger psychological literature on collective action has typically focused on injustice—or its associated emotion, anger—as an aggregate category, rather than political repression as such [24]. In addition, most research on collective action has relied on samples from Western democracies and analysed non-violent collective actions, such as protests or strikes [25]. Taken together, it remains unclear how political repression affects people's motivations to engage in collective actions against the repressors, particularly when it involves non-Western populations and anti-government *violence*.

The question of whether repression deters or motivates anti-government violence has also been a subject of extensive research within political science, sociology and economics [2,26–28]. This research has typically examined the repression–violence link at a higher level of aggregation, for example, by averaging the incidence of repression events over some periods (e.g. years) and political or geographical units (e.g. countries) and then correlating it with similarly aggregated incidence of anti-government violence. The findings of the macro research are inconsistent:

By far the most long-standing and stable influence on state repression concerns political conflict … [But] when the causal arrow is reversed and one considers research that investigates the influence of repressive behavior on dissent … the results are highly inconsistent. Sometimes the impact of repression on dissent is negative … sometimes it is positive … sometimes it is represented by an inverted U-shape … sometimes it is alternatively negative or positive … and sometimes it is nonexistent … Both findings viewed together [are referred] to as the ‘Punishment Puzzle’. [2]

Arguably, the ‘Punishment puzzle’ cannot be solved until we address the elemental question of how people, themselves, react to repression. This question remains underexplored in the voluminous macro literature, partly because the ‘data at the individual level are not easily available, particularly in the contexts of severe repression’ [28].

Important indirect evidence comes from an analysis of political loyalties in Ukraine following the Holodomor famine of 1932–1934 [29]. The authors analysed loyalty to Moscow during 1941–2014 at the level of rayons (local administrative units) and found that Ukrainian regions that experienced more famine were more loyal towards Moscow when there was a threat of retribution for anti-Soviet behaviours; however, those same regions were also more disloyal when such threats were absent. Survey data collected in eastern Ukraine in 2017 provided further support that victimization elicits revenge motivations that are acted on depending on costs: opposition to pro-Russian forces was higher among people who had a family member who died in the Holodomor; however, this association was muted for respondents in the areas most under Russian control.

A holistic account of the repression–dissent link must explain how repressed individuals are mobilized for collective action against the repressors, the tactical choices available to dissidents and repressors and many other processes and variables [2,26,28]. However, the complex path from repression to organized anti-government violence must involve people's psychological reactions to repression. Hence, a micro-level, psychological account is a crucial step in addressing the ‘Punishment puzzle’. If micro-level evidence shows that repression motivates anti-government violence, then this suggests plausible explanations for the macro findings that repression sometimes quells dissent: for example, despite causing motivations for violence, repression may also undermine the mobilization of the repressed (e.g. repressors often detain opposition leaders who are instrumental in mobilization processes). By contrast, if micro-level evidence indicates that repression deters anti-government violence, then this suggests how repression ‘works’: repression deters people from confronting the repressors (e.g. by undermining people's sense of power, as suggested by the psychological research reviewed above).

Whether repression deters or motivates anti-government violence also relates to a basic debate across the social sciences. Research shows that cost imposition or ‘punishment’ is generally an effective deterrent [30]. However, research also shows that cost imposition, if deemed unfair, elicits strong feelings of revenge, which are then pursued even at additional costs [31].

Corresponding to these debates, we examined whether political repression (unjust cost imposition) motivates people to engage in anti-government violence (costly and risky collective action). More specifically, we centred on the association between the subjective experience of political repression and behavioural intentions to participate in anti-government violence. To advance research on collective actions, we enlisted large multinational survey data from non-Western samples. Furthermore, we conducted randomized experiments. Our analyses thus aimed to maximize external and ecological validity, while at the same time addressing characteristic threats to internal validity: reverse causality and omitted variable bias.

The analyses proceed in two steps. We started with an extensive search and analysis of existing survey data that included questions about political repression and anti-government violence. These analyses relied on questions about repression available in the multinational datasets, largely reflecting moderate repression forms, such as restrictions of civil liberties. In the second step, we collected and analysed original data on a wide range of repression and violence measures by interviewing individuals from countries with widespread repression: Belarus, Venezuela and Nicaragua. As part of this second step, we also analysed random-assignment experiments that were embedded in the original three surveys.

## 2. Multinational analyses

### 2.1. Method

#### 2.1.1. Samples

**Figure 1.**
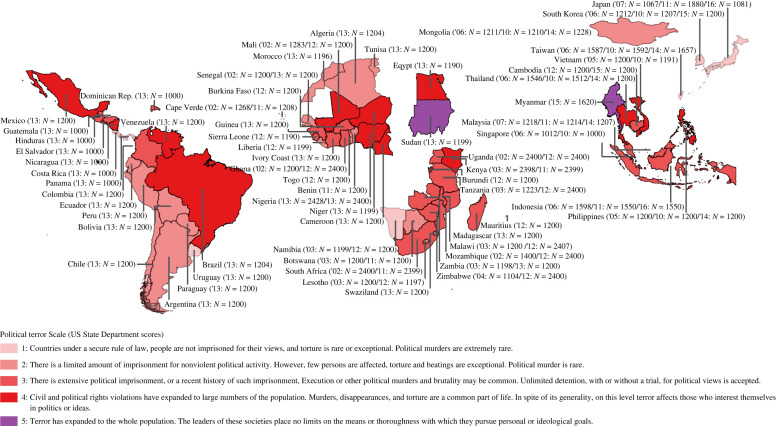
Countries, with survey years and sample sizes in parentheses, included in the multinational analyses. Colour-coding represents *The Political Terror Scale* (2018 scores) [35].

We identified six datasets that contain relevant questions about political repression and anti-government violence: Afrobarometer Round 2 (16 countries with *N*s from 1198 to 2428; total *N* = 24 301), Afrobarometer Round 5 (34 countries with *N*s from 1197 to 2407; total *N* = 51 587), Latinobarómetro 2013 (18 countries with *N*s from 1000 to 2459; total *N* = 22 663), Asianbarometer Round 2 (13 countries with *N*s from 849 to 5098; total *N* = 19 798), Asianbarometer Round 3 (13 countries with *N*s from 1000 to 3473; total *N* = 19 436) and Asianbarometer Round 4 (14 countries with *N*s from 1081 to 4068; total *N* = 19 798) [32–34]. Combined, these datasets span 109 independent samples from 67 countries. Due to limited data on some variables, our final analyses spanned 101 independent samples (*N* = 139 266) from 65 countries, which are indicated in figure 1. Electronic supplementary material, S1.1 provides further details about the multinational datasets.

#### 2.1.2. Outcomes

As the main outcome measures, we used indicators of behavioural intentions, reflecting motivations to participate in anti-government violence at the time of interview and prospectively [36]^2^. As secondary measures, we used behavioural self-reports of actual participation in anti-government violence. Both measures were binary indicators derived from questions about the use of force or violence for a political cause (table 1 provides formulations). Behavioural intentions predict—and causally relate to—actual behaviour [36,38], including participation in costly collective actions [24]. Even among frontline combatants, stated intentions and actual behaviours (e.g. sacrifices on the frontline) converge [37]. These measures also allow for assessing motivations for violence independent of opportunities: people who have not participated in violence might have taken part in it if they had opportunities, and such opportunities may be limited for reasons beyond our scope (e.g. sickness). In robustness tests, we also analysed original, ordinal versions of this variable (see electronic supplementary material, S1.6 for details). Furthermore, we measured interviewees' motivations to participate and self-reported participation in non-violent protests. For complete formulations of the questions used to derive outcome variables, reporting and endogeneity concerns, summary statistics and histograms, see electronic supplementary material, S1.2.

**Table 1.** Formulations of questions used to measure outcomes and predictors in multinational analyses.

	outcomes: political violence	predictors: perceived repression
*Afrobarometer Round 5*	*Here is a list of actions that people sometimes take as citizens. For each of these, please tell me whether you, personally, have done any of these things during the past year. If not, would you do this if you had the chance* *…* *Used force or violence for a political cause*. Answer options: *No, would never do this*; *No, but would do if had the chance*; *Yes, once or twice*; *Yes, several times*; *Yes, often*; *Don't know*.	perceived repression scale (PRS) of three items:
		*In this country, how free are you*:
		[1] *To say what you think*.
		[2] *To join any political organization you want*.
		[3] *To choose who to vote for without feeling pressured*.
		Answer options ranged from 0 = *Completely free* to 3 = *Not at all free*.
	● Behavioural intentions were coded: 0 = *No, would never do this*; 1 = *No, but would do if had the chance*.	
	● Behavioural self-reports: 0 = *No, would never do this* or *No, but would do if had the chance*; 1 = *Yes, once or twice*; *Yes, several times*; or *Yes, often*.	
*Afrobarometer Round 2*	The same as in Afrobarometer Round 5	PRS of three items:
		*We are going to compare our new government under* […] *with the former government under* […]. *Please tell me if the following things are worse or better now than they used to be or about the same:*
		[1] *Freedom to say what you think*.
		[2] *Freedom to join any political organization you want*.
		[3] *Freedom to choose who to vote for without feeling pressured*.
		Answer options ranged from 0 = *Much better* to 4 = *Much worse*.
*Latinobarómetro 2013*	*Here is a list of actions that people sometimes take as citizens. For each of these, please tell me whether you, personally, have never, once, or more than once done any of these things during the past three years* *…* *Used force or violence for a political cause*.	PRS of two items:
		*How would you evaluate the current government's performance* *…*
		[1] *regarding individual freedom of expression* *…* *and*
	● Behavioural intentions were not obtained in Latinobarómetro 2013.	[2] *regarding press freedom?*
	● Behavioural self-reports were coded: 0 = *never*; 1 = *once* or *more than once.*	Answer options ranged from 0 = *Very good* to 3 = *Very poor*.
*Asianbarometer Round 4*	*Here is a list of actions that people sometimes take as citizens. For each of these, please tell me whether you personally, have never, once, or more than once done any of these things during the past three years* *…* *Used force or violence for a political cause*.	PRS of two items:
	● Behavioural intentions were coded: 0 = *I have not done this and I would not do it regardless of the situation;* 1 = *I have not done this, but might do if something important happens in the future*.	*Now I am going to read to you a list of statements that describe how people often feel about the state of affairs in* [country name]. *Please tell me whether you* [0 = ] *strongly agree, somewhat agree, somewhat disagree, or* [3 = ] *strongly disagree with each of these statements* …
	● Behavioural self-reports: 0 = *I have not done this and I would not do it regardless of the situation* or *I have not done this, but might do if something important happens in the future;* 1 = *once* or *more than once.*	[1] *People are free to speak what they think without fear*.
		[2] *People can join any organization they like without fear*.
*Asianbarometer Round 3*	Behavioural intentions were not obtained in Asianbarometer Round 3.	As in Asianbarometer Round 4
	Behavioural self-reports were measured identically to Latinobarómetro 2013.	
*Asianbarometer Round 2*	Behavioural intentions were not obtained in Asianbarometer Round 2.	As in Asianbarometer Round 4
	Behavioural self-reports were measured identically to Latinobarómetro 2013.	

#### 2.1.3. Predictors

As the main predictor, we derived the perceived repression scale (PRS) based on a factor analysis of items in Afrobarometer Round 5 (electronic supplementary material, S1.3). We computed each interviewee's score on the scale as the mean score over three items: *In this country, how free are you*: (1) *to say what you think;* (2) *to join any political organization you want;* (3) *to choose who to vote for without feeling pressured*; reply options ranged from 0 = *completely free* to 3 = *not at all free* (Cronbach's *α* = 0.78). The other surveys included two or three similar items. For complete formulations of the questions used to derive PRS, summary statistics and histograms, see electronic supplementary material, S1.3.

#### 2.1.4. Control variables

We also analysed a set of individual-level control variables. Because post-treatment bias is a key concern for analysis of observational data, we limited control variables to a basic set: gender, age, education, subjective socioeconomic status and democratic values. In a series of robustness tests, we controlled for proxies of political activism, mobilization, government support, group-based injustice, contempt towards state authorities, political efficacy [25], dishonest responding and question comprehension. Some collective action models may consider these measures as potential mediators, rather than confounders, of the effect of repression on political violence [25]. We return to this issue in §4. For complete formulations of the questions used to derive controls, summary statistics and histograms, see electronic supplementary material, S1.4. Furthermore, drawing on the idea that the amount of selection on the observed controls in a model provides a guide to the amount of selection on unobservables, we estimated the risk of omitted-variable bias and bias-adjusted effects (see electronic supplementary material, S1.6 for details). In addition, we included a number of country-level control variables: national-level repression, political instability, violent crime, violent protests, national economic status, conflict history and national population size (see electronic supplementary material, S1.4 for details). We also used fixed-effects estimators, as well as several types of multi-level models, to account for country-level confounding (see §2.1.5).

#### 2.1.5. Modelling

The questions about anti-government violence and repression are equivalent but not identical in all six datasets; therefore, we did not merge them into one. We first analysed Afrobarometer Round 5, which has the largest coverage and the widest range of other items allowing us to assess the robustness of results to a range of alternative modelling choices. The large number of level-2 and level-1 units (i.e. countries and individuals) allowed us to conduct a comprehensive multi-level analysis, accounting for country-specific factors that potentially confound or moderate the (individual-level) associations of interest. Given binary outcomes, we used hierarchical generalized linear models with the logit link function. To aid interpretation, all covariates were normalized to range from 0 to 1. After Afrobarometer Round 5, we analysed Afrobarometer Round 2, Latinobarómetro 2013 and Asianbarometer Rounds 2–4 (Asianbarometers were merged into one dataset). See electronic supplementary material, S1.5 for detailed model specifications and multiple robustness tests.

### 2.2. Results

Hierarchical modelling identified significant and positive associations between the PRS and all outcomes in all datasets. Table 2 reports estimated average marginal effects of PRS on the probabilities of intentions to participate in violence and self-reported participation (electronic supplementary material, tables S10–S13 provide detailed estimates). According to the estimates based on Afrobarometer Round 5 (for example), a highly repressed individual (PRS = 1), compared with a non-repressed (PRS = 0), is approximately 3% points more likely to report participation in violence, and 4% points more likely to report intentions to do so. For comparison, men (compared with women) are 1% point more likely to report participation in violence (*p* < 0.001) and 2% points more likely to report intentions to do so (*p* < 0.001). Gender or sex is an established predictor of violence and aggression [39]. As shown in electronic supplementary material, figure S19, the relationship between perceived repression and intentions to engage in violence was positive in 43 samples (of 64), in 20 of which the coefficients were significant at 5% level. Analogous numbers for behavioural self-reports were 63 (of 101) and 25 (electronic supplementary material, figure S20). Additionally, we conducted analyses of cross-level interactions, exploring whether any of the above-presented level-2 controls moderated the level-1 repression–violence associations, finding no significant interaction terms (see pp. 13–14 in electronic supplementary material, for details).

**Table 2.** Intentions to participate in anti-government violence and self-reported participation as a function of the perceived repression scale (PRS). The table reports average marginal effects (with corresponding 95% CIs and *p*-values) of the PRS on the probabilities of intentions to participate in anti-government violence and self-report participation. Latinobarómetro 2013 and Asianbarometer Rounds 2 and 3 lack measures of intentions to engage in violence. Asianbarometer Rounds 2–4 contain identical questions on participation in violence and repression; therefore, these datasets were combined into one for the analysis of participation. Electronic supplementary material, tables S10–S13 provide detailed estimates. For modelling choices and robustness tests, see electronic supplementary material, S1.5.

		behavioural intentions to participate in anti-government violence	behavioural self-reports of participation in anti-government violence
*Afrobarometer Round 5* (2011–2013)	34 samples, *N* = 51 587	**4.1%**	**3.3%**
		95% CI = (2.9–5.1)	95% CI = (2.3–4.2)
		*p* < 0.001	*p* < 0.001
*Afrobarometer Round 2* (2002–2004)	16 samples, *N* = 24 301	**5.8%**	**4.1%**
		95% CI = (3.7–8.0)	95% CI = (2.6–5.6)
		*p* < 0.001	*p* < 0.001
*Latinobarómetro 2013* (2013)	18 samples, *N* = 20 204	—	**1.6%**
			95% CI = (0.2–2.9)
			*p* < 0.001
*Asianbarometer Round 4* (2014–2016)	11 samples, *N* = 14 360	**3.9%**	**0.7%**
		95% CI = (1.1–6.6)	95% CI = (0.2–1.3)
		*p* = 0.006	*p* = 0.013
*Asianbarometer Round 3* (2010–2012)	12 samples, *N* = 15 963	—	
*Asianbarometer Round 2* (2004–2008)	10 samples, *N* = 12 851	—	

We focused on anti-government violence; however, we also explored how repression affects motivations to participate in non-violent collective actions, namely, protests. We identified significant and positive associations between PRS and intentions to participate in protests and self-reported participation in the samples of Afrobarometer Round 5 and Asianbarometers Rounds 2–4, but not in the samples of Afrobarometer Round 2 and Latinobarómetro 2013 (electronic supplementary material, tables S14–S17 provide detailed estimates).

## 3. Single-country analyses

### 3.1. Method

#### 3.1.1. Samples

We first conducted a pilot survey of citizens of Belarus, a state with a robust security apparatus. We assumed that citizens of Belarus would have a realistic grasp of risks involved in violence against a repressive regime, and hence report realistic behavioural intentions. Following our discussions with area experts, we concluded that conducting a survey about repression and anti-government violence would be unfeasible in Belarus. Therefore, using an unusual situation, we interviewed Belarusians in Lithuania, which hosts the European Humanities University (EHU), relocated from Belarus after a forced closure by the regime. The interviewees (*N* = 386) were students who either resided in Belarus and visited the university for exams or resided in Lithuania for the whole study period. Hence, the EHU sample provided a unique opportunity to pilot and develop our survey instrument in close contact with those who have first-hand experience with repression. We administered the survey in Russian in February and March 2017 and collected 386 fully or partly completed questionnaires (nearly half of all university's students).

We then searched for opportunities to collect large probability samples via online surveys in countries experiencing both repression and anti-government violence. We assumed that interviewees from these countries would also report realistic behavioural intentions. In addition, we expected such samples to contain actual participants in violence. We first interviewed citizens of Venezuela (*N* = 1000), which experienced anti-government protests during the survey period (September 2017), with hundreds of deaths and thousands injured. Five months prior to our survey, up to six million people (one in five of the population) reportedly participated in the nationwide Mother of all Marches [40]. In the Venezuelan sample, 52.7% of interviewees (527 of 1000) reported participation in protests, and 4.5% (45) in violence, over the last year. We then conducted a pre-registered (link to the pre-registration at OSF: https://osf.io/5k3d6) replication study in Nicaragua (*N* = 1574), which also experienced violent large-scale protests prior to and during the survey period (October 2018). In this sample, 29.1% of interviewees (458 of 1574) reported participation in protests and 1.8% (28) reported participation in violence. Both surveys were administered in Spanish via survey agency YouGov, and quota-sampled for age, gender and geography to obtain nationally representative samples of online populations. See electronic supplementary material, S2.1 for detailed description of data collection procedures.

#### 3.1.2. Outcomes

The survey at EHU only measured behavioural intentions to engage in violence, because there were no salient events of anti-government violence in Belarus during or prior to the survey. We used a 2-item scale, answered on a 7-point Likert-type scale from 0 = *Would never do this* to 6 = *Would certainly do this* (Cronbach's *α* = 0*.*79):

1. Tell a friend that, in some circumstances, it is justified to use violence for a political cause.
2. Use force or violence for a political cause.

To assess whether interviewees under-reported their true intentions (e.g. due to fears of reprisals), we also measured intentions with an indirect measure of support for violence against the actual government of Belarus, derived from a double list experiment [41], an item-count technique [42]. A list experiment allows estimating agreement with statements about sensitive topics without asking individual interviewees to explicitly indicate agreement with specific statements, which probably decreases the risk of dishonest or socially desirable responding. The double list experiment is a simple extension of the standard list experiment aimed at increasing estimate accuracy, by having each participant complete two list experiments (lists A and B; see electronic supplementary material, S2.2). We found little evidence of under-reporting (see electronic supplementary material, S2.6); therefore, our main analyses relied on the standard measures as described above.

In Venezuela and Nicaragua, we measured both behavioural intentions to engage in violence and self-reported participation (as well as intentions to engage and self-reported engagement in protests), since these populations experienced large-scale and violent anti-government protests during the survey period. For comparability with multinational analyses, we used identical formulations as in Afrobarometer Rounds 2 and 5. In addition, we obtained an alternative, validated measure, the radicalism intention scale (RIS) [43]. This scale consists of four items (e.g. *I would participate in a public protest against oppression of my group even if I thought the protest might turn violent*; from 0 = *Very unlikely* to 6 = *Very likely*). The surveys in Venezuela and Nicaragua also included a double list experiment to measure intentions to engage in violence, and another 4-item scale measuring intentions to participate in violence after an experimental vignette (see §3.1.5).

#### 3.1.3. Predictors

**Figure 2.**
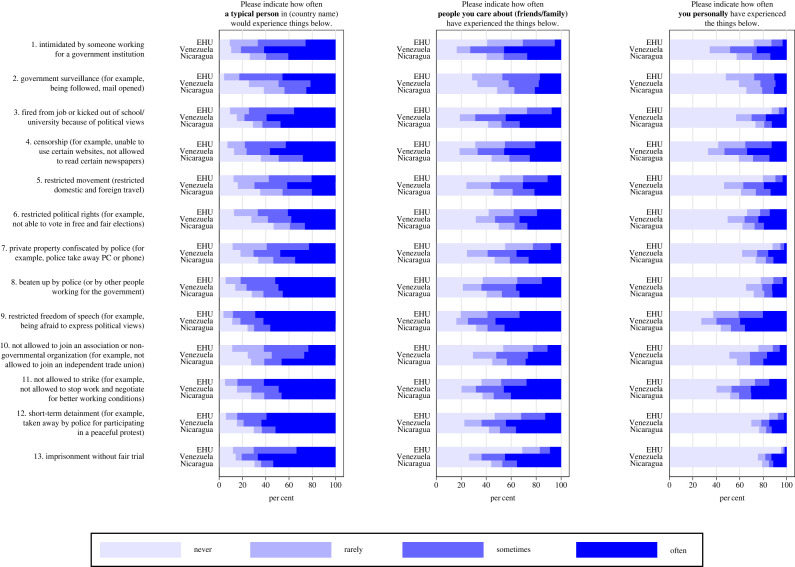
Political repression of Belarusians, Venezuelans and Nicaraguans.

Repression was measured with a 39-item instrument that we developed based on existing literature and on our pilot study, following multiple rounds of discussions with the staff and students of the EHU. We first presented an explanation of what we considered political repression: *In some countries, the government (or people working for the government) might use intimidation or violence against citizens. The government (or people working for the government) might use intimidation or violence to stop people from participating in certain activities or having certain political views*. Then, followed a list of 13 events for which we asked the following question: *Below are several things that may or may not happen to a person. Please indicate how often a typical person in Belarus would experience the things below*; 0 = *Never*, 1 = *Rarely*, 2 = *Sometimes*, 3 = *Often*. The 13 events were based on the definition and examples of repression presented in existing work [2,26]. We also consulted with area experts, including the EHU staff, to include examples of repression that might have happened to individuals in the sample population. The formulations of the 13 items are shown in figure 2. After reporting their personal experience, the interviewees were asked to answer the same 13 items considering their friends and family (*Please indicate how often people you care about (your friends and family) have experienced the things below*), and themselves personally (*Please indicate how often you personally have experienced the things below*).

All samples reported widespread repression. For example, roughly every eighth (11.97%) EHU student reported property confiscation by police and nearly half (44.77%) reported such confiscation among friends and family. Every third to fourth in Venezuela (34.56%) and Nicaragua (28.03%) reported having been physically beaten by police or other people working for the government.

For the regression analyses, we computed six repression variables (electronic supplementary material, table S7 and figure S14 provide summary statistics and histograms):

1. RS (aggregate): an aggregate repression scale, the average response over all 39 items (*α* = 0.94);
2. RS (typical people): repression of typical people, the average response over the 13 items about a typical person (*α* = 0.91);
3. RS (friends/family): repression of friends and family, the average response over the 13 items about friends and family (*α* = 0.90);
4. RS (self): repression of the self, the average response over the 13 items about interviewees' own experience of repression (*α* = 0.86);
5. RS (high-intensity): high-intensity repression, the average of the four high-intensity repression items (i.e. intimidated by someone working for the government, beaten up by police, short-term detainment, imprisonment without fair trial) across typical people, friends and family and self (12 items, *α* = 0.84);
6. RS (low-intensity): low-intensity repression, the average of the remaining nine low-intensity repression items across typical people, friends and family and self (27 items, *α* = 0.92).

We used the same questions to measure repression in the surveys in Venezuela (all *α* > 0.91) and Nicaragua (all *α* > 0.94). Electronic supplementary material, tables S8 and S9 and figures S16 and S18 provide summary statistics and histograms. The surveys in Venezuela and Nicaragua also asked about experience of torture. For comparability with the Belarusian sample, we excluded the torture item from the repression scales in the Venezuelan and Nicaraguan samples.

#### 3.1.4. Control variables

For analyses of observational data, we obtained the same basic set of control variables in Belarus, Venezuela and Nicaragua: gender, age, education (not measured in the Belarusian sample), socioeconomic status and democratic values (see electronic supplementary material, S2.4). For the experimental analyses (see below), we did not control for any additional variables.

#### 3.1.5. Experimental manipulations

Repression cannot be experimentally administered; therefore, drawing on existing experimental paradigms [44,45], we developed designs to temporarily induce thoughts about repression. We assumed that thoughts or memories about repression evoke psychological responses that resemble those evoked by the actual experience. The pilot study at the EHU included a guided recall task that asked interviewees to think of the Belarusian political situation and that varied the recall instructions between interviewees. However, this task suffered from non-compliance (electronic supplementary material, S2.5). Therefore, we developed an alternative design for the surveys in Venezuela and Nicaragua. Specifically, we used the set of 39 repression items itself as a manipulation instrument. Replying to the questions, as listed in figure 2, about experienced repression in the past (or observed in a given country) should naturally induce thoughts about repression. This experimental procedure builds on existing questions-as-treatment designs, where treatment questions are formulated and located immediately before outcome items to transiently increase the saliency of particular thoughts (e.g. *How close do you feel to your ethnic or racial group?* to manipulate saliency of ethnic identity) [45–49]. Thus, in the surveys in Venezuela and Nicaragua, half of the interviewees first received the repression items (experimental condition), immediately followed by the questions measuring intentions for anti-government violence (*n*s = 492 and 771 in Venezuela and Nicaragua, respectively). The other half of the interviewees started the survey with the questions about anti-government violence (*n*s = 508 and 803 in Venezuela and Nicaragua, respectively).

The surveys in Venezuela and Nicaragua also included experiments to measure the effect of governments' remedial actions on citizens’ intentions to participate in violence. Both surveys included vignettes describing a hypothetical, but realistic, situation familiar to our interviewees: a conflict between a newly elected government and opposition, during which a protester was killed by police. Half of the interviewees received a text with a conciliatory gesture (*n*s = 497 and 774 in Venezuela and in Nicaragua, respectively):

Imagine that there were presidential elections in Venezuela [/Nicaragua] last week and the candidate that you oppose has claimed to be the new president of Venezuela [/Nicaragua]. All week there were big protests in the large cities by people like you. People like you—who oppose the new president—think that the elections were not fair and do not accept the new president. During one of the protests, a protester was killed by the police. Videos that circulate on the internet show a police officer hitting the protestor multiple times with a stick. The protestor was lying on the ground and did not have any weapons. Today the new president issued a statement that he will not tolerate police brutality and that there would be a comprehensive investigation of the event.

For the other half of the interviewees (*n*s = 503 and 800 in Venezuela and in Nicaragua, respectively), the last sentence of the vignette was different so that it did not include a conciliatory gesture (i.e. *Today the new president issued a statement that the authenticity of the video could not be established and that there would be no further investigation of the event*).

In Venezuela, the vignette was followed by two questions that served as manipulation checks, the first measuring perceived concern for well-being of citizens, the second measuring anger about the event (electronic supplementary material, S2.5). Responses to these questions showed that the remedial action significantly increased perceived concern for well-being of citizens, *b* = 1.94, s.e. = 0.12, *t*_938_ = 15.78, *p* < 0.001 but did not significantly influence anger about the killing, *b* = 0.14, s.e. = 0.08, *t*_959_ = 1.68, *p* = 0.093.

Then, there followed four items measuring intentions to engage in violence. The interviewees were asked to indicate intentions to respond to the killing in four ways (answered on a 7-point scale from 0 = *Would never do this* to 6 = *Would certainly do this*):

1. Tell a friend that it is justified to use violence to oppose the new president.
2. Help members of a violent political movement that opposes the new president.
3. Join a violent political movement that opposes the new president.
4. Use force or violence as part of fighting the new president.

In the Nicaragua survey, the vignette was followed by the question about concern for well-being of citizens, and the remedial action increased perceived concern for the well-being of citizens, *b* = 1.90, s.e. = 0.11, *t*_1362_ = 16.61, *p* < 0.001 (the item about anger was not included). The survey then included the same four items measuring intentions to engage in violence, with the following variation in the instructions.

It is possible that interviewees, while reporting their intentions in a survey questionnaire, may not seriously consider the risks involved in actual political violence. Therefore, the vignette experiment in Nicaragua included another manipulation. Half of the interviewees were given the same instructions as in the Venezuela survey. For the other half (*n* = 808), the instruction (before the item about violent intentions) contained an additional sentence, which read: *Please consider the risks involved in the indicated activities, for example, injury, capture or imprisonment*. This additional manipulation enabled a test of whether consideration of risks associated with anti-government violence influenced interviewees reported intentions. For further details on experimental designs, see electronic supplementary material, S2.5.

#### 3.1.6. Modelling

We analysed continuous outcomes (the measure of behavioural intentions in the Belarusian sample and radicalism intention scale in the Venezuelan and Nicaraguan samples) using standard ordinary least squares (OLS) regressions, and binary outcomes (the Afrobarometer-based measures of behavioural intentions and self-reports in the Venezuelan and Nicaraguan samples) using standard logit regressions. We analysed each sample separately. Analyses of observational data conditioned on the basic set of controls as described above. The random-assignment experiments were analysed with simple regressions, i.e. without conditioning on any controls.

### 3.2. Results

**Figure 3.**
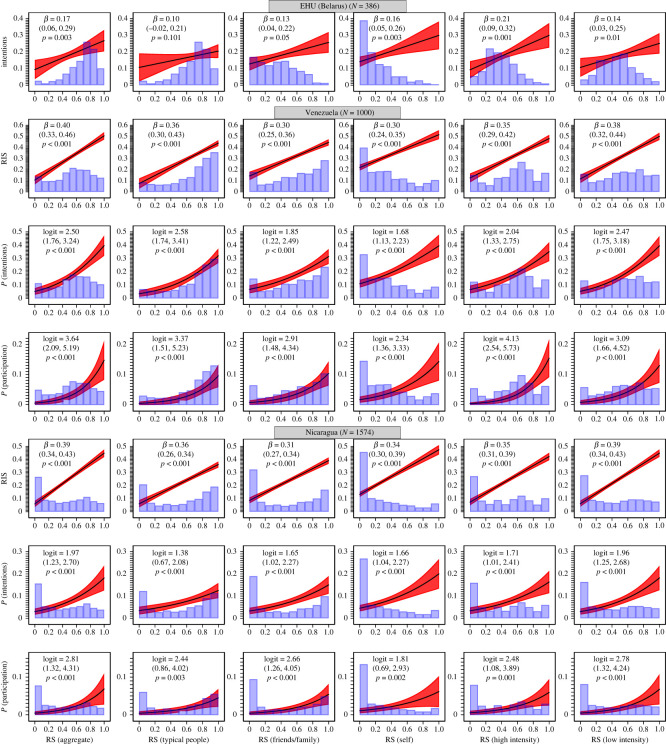
Predicted values and probabilities (with 95% CIs) of radicalism intentions (radicalism intention scale, RIS), intentions to participate in political violence, and self-reported participation in political violence as a function of repression scales, RS. Predictors and outcomes are 0–1 normalized.

Repression significantly varied with intentions to engage in violence and self-reported participation. Figure 3 reports the main results (all variables are normalized to range from 0 to 1) (electronic supplementary material, tables S26–S31, S34, S35, S38–S41, S44, S45 provide detailed estimates). An aggregate 39-item repression scale, RS (aggregate), significantly and positively predicted all outcomes in all samples. The subtypes of repression by the target of repression also significantly predicted all outcomes in all samples, save for RS (typical people) in the Belarusian sample. We also analysed high-intensity repression (violence/intimidation) and low-intensity repression (restrictions of civil liberties). Both significantly predicted all outcomes in all samples. The analyses also revealed substantial effect sizes. For example, a highly repressed citizen of Nicaragua, RS (aggregate) = 1, compared with a non-repressed citizen, RS (aggregate) = 0, was considerably more likely to report participation in violence, 7% versus 0.5%, and intentions to participate in violence, 18% versus 3%. Analogous probabilities for Venezuela were: 15% versus 0.5% and 40% versus 5%.

Repression also significantly varied with motivations to engage in non-violent collective actions. The aggregate repression scale, and all the repression sub-scales (typical people, friends/family, self, high intensity and low intensity), significantly and positively predicted intentions to engage in protests and self-reported participation in Venezuela (electronic supplementary material, tables S32, S33, S36, S37) and Nicaragua (electronic supplementary material, tables S42, S43, S46, S47) (in the Belarusian sample, we only measured violent outcomes).

Turning to random-assignment experiments: in Venezuela, considering repression increased intentions to participate in violence from 16% to 22% (*p* = 0.017) and radicalism intentions—on 0–1 scale—from 0.289 to 0.348 (*p* = 0.001). Estimates in Nicaragua were 5% versus 10% (*p* < 0.001) and 0.200 versus 0.243 (*p* = 0.003), respectively.

As noted in §3.1.5, we also assessed a corollary prediction that remedying repression reduces violent intentions. In Venezuela, remedial actions by state authorities reduced intentions to engage in violence—on 0–1 scale—from 0.197 to 0.161 (*p* = 0.039); and in Nicaragua, from 0.104 to 0.066 (*p* < 0.001).

Finally, we found that priming the risks of injury, capture and imprisonment prior to reporting violence intentions did not influence the reported intentions for anti-government violence, *b* = 0.01, s.e. = 0.01, *t*_1488_ = 0.69, *p* = 0.492. This suggests that even without an explicit reminder of the risks, interviewees considered the risks involved in violence while reporting behavioural intentions.

## 4. Discussion

State authorities use repression to subdue political opposition. Our studies indicate, however, that such coercion increases motivations for anti-government violence. Whether violence indeed breaks out depends on multiple other processes, some of which are also influenced by repression. Aside from citizen's motivations, repression influences the opposition's opportunities for mobilization. Jailing opposition activists, for example, can cause anger among citizens but, at the same time, undermine their ability to mobilize for collective action against the repressors. These group- or state-level processes have been extensively examined within political science, economics and sociology [26], and are beyond our study's scope. We focused on a more elemental, micro-level question of whether repression decreases or increases citizens' *motivations* for violence. We found that repression motivates anti-government violence. This implies that if such violence does not occur, then this is probably due to undermined mobilization opportunities. Hence, repressive tactics may quell the opposition in the short term, but at the cost of alienating ordinary citizens, who—given the opportunity—will more likely resort to violence.

Corroborating this, research has documented a relationship between popular support for political violence and actual events of such violence. Using Afrobarometer data, Linke *et al*. [50] tested whether average levels of people's endorsement of political violence in administrative units at certain periods predicted political violence events in those units at subsequent periods, finding a positive association. Linke *et al*. focused on attitudinal support for/endorsement of political violence. Attitudinal support for violence is not synonymous with intentions to engage in violence (i.e. what we measured), but the two are closely related. In standard collective action models in social psychology, attitudinal support for/endorsement of collective action is considered as the first step, preceding motivations or intentions, in the processes leading to participation [24,51]. In these models, like in other models of attitude–behaviour relations [38,52], intentions to engage in collective actions are considered as closer proxies of actual participation, compared with mere attitudes. Hence, if attitudes towards violence predict actual incidence of violence—as shown by Linke *et al*.—then intentions should be an even stronger predictor. Whether this is indeed true is an interesting empirical question for further research.

More generally, our findings contribute to an emerging literature across the behavioural sciences on the psychology that gives rise to feelings of revenge [31]. Theoretical accounts conceptualize revenge, or retaliatory aggression, as a negotiation strategy designed to upregulate the weight of the retaliator's welfare in the social decisions of others [31,53]. By imposing costs on individuals who put too little weight on the retaliator's welfare, revenge serves as an incentive for targets and bystanders to place more weight on the welfare of the agent. Over evolutionary history, organisms that could not defend their fitness interests in this way faced significant costs; therefore, today we observe revenge behaviour not only among humans but virtually across all social species [53,54]. While our studies did not directly measure feelings of revenge or its associated psychological processes, our results are consistent with the notion that repression—as unjust cost imposition—motivates retaliation, despite its risks and costs.

Our robustness tests included a number of proxies of variables highlighted in social psychological research on normative and non-normative collective actions [25], such as perceived group-based injustice and contempt towards state authorities. In our analyses, we considered these measures as potential confounders of the repression–violence associations. For example, our measure of ethnic discrimination or perceived group-based injustice may capture economic injustices (e.g. due to group-based income inequality), which—studies suggest [55]—predict support for political violence. If such inequality-related injustices correlate with repression (politically repressed groups are also likely to be disadvantaged economically), then our identified associations between political repression and violence may in part (spuriously) reflect inequality–violence associations. However, alternative interpretations are possible, including those that consider our proxy of group-based injustice as a mediator of the repression effects on political violence [27]. Typically, such conceptualizations of injustice-related variables as mediators emphasize their affective side, such as anger or contempt. However, since our proxies of perceived injustice and other variables did not explicitly tap emotions, we did not assess them in formal mediation analyses. Whether constituting confounders or mediators, these proxies—included as controls in regression models—did not notably attenuate the coefficients representing the repression–violence associations.

Importantly, our findings concur with recent research on collective actions in repressive contexts, showing that perceived risks of government sanctions increase—not decrease—motivations to engage in collective actions [20,21]. In particular, Ayanian *et al*. [21] surveyed protesters in Russia, Ukraine, Hong Kong and Turkey. The authors examined how intentions to participate in future non-violent protests were influenced by perceived risks and several mediating variables. The results showed that perceiving more risk was associated with stronger intentions to participate in future protests, and that this relation was mediated by outrage, efficacy and several other variables. The measure of perceived risk that Ayanian *et al*. used probably also captured perceptions of being repressed (e.g. estimates of the likelihood that protesting leads to being detained). Hence, this research suggests that perceptions of potential repression increase motivations to participate in future protests, at least among individuals who already participated in protest. Our studies show consistent results. Using other measures of repression (which focus on experience of repression, not perceived risk) and intentions to engage in both non-violent and violent dissent, as well as broader samples and experimental manipulations, we also found that repression motivates rather than deters dissent.

### 4.1. Limitations

Research on political repression and violence faces several design constraints. First, we cannot experimentally administer the key predictor of interest (i.e. experience of repression). To address this challenge, we developed designs that aimed to manipulate thoughts about repression. These designs draw on established experimental paradigms and invoke the assumption that thoughts or memories about repression temporarily induce responses resembling those evoked by the actual experience. However, we did not assess this assumption empirically. Compared with the actual experience, thoughts about repression may evoke weaker fear; in such a scenario, our analyses would overestimate the effects of repression on violence. However, thoughts about repression may also evoke weaker anger; in this scenario, our analyses would underestimate the effects of repression on violence. We are not aware of any approaches to assess these scenarios empirically, as we cannot experimentally manipulate experience of repression. Finally, although the results of the experiments support a causal effect in one direction (repression causing motivations for violence), this does not imply the absence of a causal path in the opposite direction (motivations for violence causing perceptions of repression). Future work may examine whether the relation between violent collective action and repression is bidirectional.

Second, naturalistic observation of the key outcome of interest (i.e. participation in violence) is very difficult. We can measure changes in attitudes and intentions towards violence following an experiment, but we can hardly observe whether interviewees subsequently engage in real-world violence. To address this challenge, we relied on measures of behavioural intentions. The use of such measures as proxies of behaviour is standard in psychology [36,38]. However, although studies suggest that behavioural intentions predict participation in non-violent collective actions (e.g. protests) [24], the evidence is more limited when it comes to collective violence. Instead of using intentions as proxies of behaviour, some studies used interviews of frontline combatants about their behaviour in armed conflicts [37]. Such interviews may generate more valid evidence than surveys of behavioural intentions. However, interviewing combatants in armed conflicts faces major logistical, security and ethical challenges, limiting such research to particular countries and small samples. Multinational studies suggest that predictors of participation in political violence vary greatly across countries [56]; hence, research based on single-country samples may be confounded or moderated by country-specific characteristics. Importantly, combatant interviews confirm that stated behavioural intentions converge with actual behaviour, providing a rare piece of evidence on the validity of behavioural intentions in the study of political violence [37]. Measures of behavioural intentions also allow assessing people's motivations to take part in violence independent of opportunities: people who have not participated in violence could have taken part in it if they had an opportunity to do so. In addition, given that intentions to partake in violence does not constitute actual violence, such measures may also suffer less from reporting bias, compared, for example, with behavioural self-reports. In addition to behavioural intentions or behavioural self-reports, scholars have recently started developing virtual simulations of combat, using professional soldiers as participants [57]. However, at present, all types of evidence on participation in political violence remain scarce and hence constitute prospective avenues for future research.

Third, given the sensitive nature of our research subject, we potentially faced non-response problems. To address this issue, we (i) took *a priori* measures to prevent non-response bias and (ii) analysed list experiments, non-response rates and non-responders, which provided little evidence of such bias. We estimate that non-response bias, if present, has probably generated conservative estimates in our case (for details, see electronic supplementary material, S2.6).

Finally, state authorities in repressive regimes may not allow surveying citizens about repression and anti-government violence. Some research therefore relies on convenience samples (e.g. students) and vignettes with hypothetical scenarios or economic games simulating some aspect of political violence. However, individuals exposed to such simulations rarely have first-hand experience with repression or political violence and hence a limited grasp of real-world injustices, risks and costs involved in violence against repressive regimes. To address this challenge, we searched for opportunities to interview individuals from repressive regimes experiencing widespread violence. The European Humanities University, a university relocated to a democratic country, provided us with a unique opportunity to pilot and develop our survey instrument in close contact with those who had first-hand experience with repression. Subsequently, we searched for opportunities to collect larger surveys in countries experiencing repression and political violence. Our surveyed samples probably contained substantial numbers of individuals who directly engaged in anti-government protests or at least observed them in their immediate environments. We assumed that such interviewees would provide realistic behavioural intentions regarding participation in violence. Still, while replying to survey questionnaires, interviewees may evaluate risks and costs differently compared with when they deliberate on actual engagement in anti-government behaviour. To address this challenge, we designed an additional experiment that explicitly asked interviewees to consider risks and costs involved in anti-government violence. We found that such priming did not influence interviewee's replies, substantiating our measures of behavioural intentions. However, whether interviewees consider risks similarly while stating behavioural intentions in survey questionnaires and while considering actual engagement in violence is another important subject for future research.

### 4.2. Conclusion

Political repression involves abuse of human rights and hence is normatively undesirable. This notwithstanding, state authorities, across the globe (figure 1), continue to use repression, expecting to maintain their political power. However, our studies suggest that repression gives rise to revenge motivations among the citizens they repress. Hence, in addition to normative considerations, our research points to a strategic rationale for governments to halt repression and, instead, use non-coercive means to address opposition. Reducing repression probably enhances—not undermines—political stability and hence may be in the long-term interest of governments.

## Data Availability

We conducted two types of analyses: multinational and single-country. The data used in the multinational analyses are secondary (i.e. collected by other projects) and available via http://www.afrobarometer.org/ (no subscription required; freely available data for public use), https://www.latinobarometro.org/ (no subscription required; freely available data for public use) and http://www.asianbarometer.org/ (freely available data for public use; requires an application prior to use). The data used in single-country analyses—EHU (Belarus), Venezuela and Nicaragua—are original (i.e. collected by our project's team) and freely accessible at a public repository (Open Science Framework): https://osf.io/nyx7u. The code script for all analyses (multinational and single country) is also freely available at a public repository (Open Science Framework): https://osf.io/nyx7u. The data are provided in electronic supplementary material [58].

## Footnotes

1 This goal of repression is included in an established conceptualization of political repression as ‘the actual or threatened use of physical sanctions against an individual or organization, within the territorial jurisdiction of the state, for the purpose of imposing a cost on the target as well as deterring specific activities and/or beliefs perceived to be challenging to government personnel, practices or institutions’ [2].

2 We follow Sheeran's [36, p. 2] definition of behavioural intentions, as ‘…people's decisions to perform particular actions. Intentions can be inferred from participants' responses that have the form, ‘I intend to do X’, ‘I plan to do X’ or ‘I will do X’. In psychological terms, a behavioural intention indexes a person's motivation to perform a behaviour. That is, behavioural intentions encompass both the direction (to do X versus not to do X) and the intensity (e.g. how much time and effort the person is prepared to expend in order to do X) of a decision’.
